# Innovations in Doctoral Training and Research on Tinnitus: The European School on Interdisciplinary Tinnitus Research (ESIT) Perspective

**DOI:** 10.3389/fnagi.2017.00447

**Published:** 2018-01-12

**Authors:** Winfried Schlee, Deborah A. Hall, Barbara Canlon, Rilana F. F. Cima, Emile de Kleine, Franz Hauck, Alex Huber, Silvano Gallus, Tobias Kleinjung, Theodore Kypraios, Berthold Langguth, José A. Lopez-Escamez, Alessandra Lugo, Martin Meyer, Marzena Mielczarek, Arnaud Norena, Flurin Pfiffner, Rüdiger C. Pryss, Manfred Reichert, Teresa Requena, Martin Schecklmann, Pim van Dijk, Paul van de Heyning, Nathan Weisz, Christopher R. Cederroth

**Affiliations:** ^1^Department of Psychiatry and Psychotherapy of the University of Regensburg at Bezirksklinikum Regensburg, University of Regensburg, Regensburg, Germany; ^2^NIHR Nottingham Hearing Biomedical Research Centre, Nottingham, United Kingdom; ^3^Otology and Hearing Group, Division of Clinical Neuroscience, School of Medicine, University of Nottingham, Nottingham, United Kingdom; ^4^Section of Experimental Audiology, Department of Physiology and Pharmacology, Karolinska Institutet, Stockholm, Sweden; ^5^Clinical Psychological Science, Faculty of Psychology and Neuroscience, Maastricht University, Maastricht, Netherlands; ^6^Department of Otorhinolaryngology/Head and Neck Surgery, University Medical Center Groningen, University of Groningen, Groningen, Netherlands; ^7^Institute of Distributed Systems, Ulm University, Ulm, Germany; ^8^Department of Otorhinolaryngology, Head and Neck Surgery, University Hospital of Zurich, University of Zurich, Switzerland; ^9^Department of Environmental Health Sciences, IRCCS - Istituto di Ricerche Farmacologiche Mario Negri, Milan, Italy; ^10^Otology and Neurotology Group, Department of Genomic Medicine, Centro Pfizer – Universidad de Granada – Junta de Andalucía de Genómica e Investigación Oncológica (GENYO), Granada, Spain; ^11^Department of Otolaryngology, Instituto de Investigación Biosanitaria ibs.GRANADA, Hospital Universitario Virgen de las Nieves, Universidad de Granada, Granada, Spain; ^12^Neuroplasticity and Learning in the Healthy Aging Brain (HAB LAB), Department of Psychology, University of Zurich, Zurich, Switzerland; ^13^Department of Otolaryngology, Laryngological Oncology, Audiology, and Phoniatrics, Medical University of Lodz, Lodz, Poland; ^14^Centre National de la Recherche Scientifique, Aix-Marseille University, Marseille, France; ^15^Institute of Databases and Information Systems, Ulm University, Ulm, Germany; ^16^Department of ORL and Head and Neck Surgery, Antwerp University Hospital, University of Antwerp, Antwerp, Belgium; ^17^Division of Physiological Psychology, Centre for Cognitive Neuroscience, University of Salzburg, Salzburg, Austria

**Keywords:** tinnitus, education, medical, hearing, PhD studentship, heterogeneity of tinnitus

## Abstract

Tinnitus is a common medical condition which interfaces many different disciplines, yet it is not a priority for any individual discipline. A change in its scientific understanding and clinical management requires a shift toward multidisciplinary cooperation, not only in research but also in training. The European School for Interdisciplinary Tinnitus research (ESIT) brings together a unique multidisciplinary consortium of clinical practitioners, academic researchers, commercial partners, patient organizations, and public health experts to conduct innovative research and train the next generation of tinnitus researchers. ESIT supports fundamental science and clinical research projects in order to: (1) advancing new treatment solutions for tinnitus, (2) improving existing treatment paradigms, (3) developing innovative research methods, (4) performing genetic studies on, (5) collecting epidemiological data to create new knowledge about prevalence and risk factors, (6) establishing a pan-European data resource. All research projects involve inter-sectoral partnerships through practical training, quite unlike anything that can be offered by any single university alone. Likewise, the postgraduate training curriculum fosters a deep knowledge about tinnitus whilst nurturing transferable competencies in personal qualities and approaches needed to be an effective researcher, knowledge of the standards, requirements and professionalism to do research, and skills to work with others and to ensure the wider impact of research. ESIT is the seed for future generations of creative, entrepreneurial, and innovative researchers, trained to master the upcoming challenges in the tinnitus field, to implement sustained changes in prevention and clinical management of tinnitus, and to shape doctoral education in tinnitus for the future.

## Background

Tinnitus is a condition associated with a continuous auditory percept in the ears or head and can arise as a symptom of many different medical disorders. Assuming a conservative tinnitus prevalence of 10% (1% of severe tinnitus; McCormack et al., 2016) for the 425 million adults living within the European Union (EU), tinnitus affects more than 42 million citizens and is experienced as a severe problem by more than 4 million. Moreover, incidence of new cases is expected to grow over the next few decades (Nondahl et al., 2002, 2010). Although much progress has been made in understanding the pathophysiology (Langguth et al., 2013; Elgoyhen et al., 2015), tinnitus remains a scientific and clinical enigma (Baguley et al., 2013). Unfortunately, tinnitus remains an unmet clinical need and complaining patients are often told “to live with it” (Cederroth et al., 2013). The condition is very common and of varying severity, but the fundamental mechanisms of tinnitus are still incompletely understood. Although not all individuals are unduly troubled, many find the disorder life-changing. In cases with severe tinnitus, mental disorders, and symptoms such as anxiety, depression, insomnia, and concentration problems can impair quality of life often to a level that leads to sick leave and disability pension (Friberg et al., 2012). Therefore, severe tinnitus contributes to a substantial cost to health care and to society at large. In the Netherlands alone, the economic burden of tinnitus is estimated at up to €10.8 billion, with the greater impact being related to socio-economic factors (Maes et al., 2013). The health care costs are enormous. In England there are 750,000 medical consultations yearly with the primary complaint of tinnitus (El-Shunnar et al., 2011).

There is no licensed pharmacological therapy and there is little high quality evidence for the success of palliative management strategies. The Cochrane Library currently lists 9 completed systematic reviews on different tinnitus treatments; namely Tinnitus Retraining Therapy (TRT), Cognitive Behavioral Therapy (CBT), hyperbaric oxygen therapy, sound therapy (masking), hearing aids, repetitive transcranial magnetic stimulation (rTMS), ginkgo biloba, anticonvulsants, and antidepressants (Phillips and McFerran, 2010; Hoekstra et al., 2011; Meng et al., 2011; Baldo et al., 2012; Bennett et al., 2012; Hobson et al., 2012; Hilton et al., 2013; Espinosa-Sánchez et al., 2014; Hoare et al., 2014; Person et al., 2016). However, no uniformly effective treatment for tinnitus has yet been identified. Two main reasons have been discussed: (1) methodological limitations in study design with a paucity of Randomized Controlled Trials (RCTs) and no consensus about what and how to measure therapeutic outcome (Hall et al., 2016), and (2) a large heterogeneity in the patient population with respect to etiology, genetic, and clinical phenotype (Elgoyhen et al., 2015).

Tinnitus is a symptom rather than a distinct disease, and its multivariate manifestations can be subtyped according to various dimensions such as its etiology, time since onset, perceptual characteristics (i.e., pitch, loudness, location, and temporal dynamics), perceived emotional distress, and comorbidities. Progress in scientific understanding and clinical management needs to first address this heterogeneity by identifying scientifically and clinically meaningful subtypes of the condition. Subtyping can then guide the definition of relevant inclusion/exclusion criteria in clinical research and of stratification variables for allocating patients to different intervention groups in RCTS. Subtyping can also guide more sophisticated methods of multivariate data analysis than have been applied hitherto. Current knowledge about etiology, perceptual characteristics, and the neurobiological correlates of tinnitus is not sufficient to enable effective subtyping (Lopez-Escamez et al., 2016). These problems are indicating the need for new approaches.

## An interdisciplinary approach is critical

Across the EU, no single healthcare system, research organization, or commercial enterprise has an adequate coverage of all relevant issues related to tinnitus and this has resulted in a patchwork of approaches without any coherent framework. Clinically speaking, tinnitus is managed by a variety of different practitioners including general practitioners, otologists, audiologists, psychologists, psychiatrists, neurologists, physical therapists, and dentists (Hall et al., 2011). Academically speaking, tinnitus is of interest to animal neurophysiologists, neuroscientists, epidemiologists, geneticists, trialists, biostatisticians, biomedical engineers, software engineers, and data mining experts. The European School on Interdisciplinary Tinnitus Research (ESIT) uniquely fosters an environment where knowledge and ideas are shared beyond current sectoral borders. The ESIT project provides an international interdisciplinary network of experts from these relevant disciplines working toward a coordinated approach to tinnitus research. Such a partnership is an essential part of the project. It will support data sharing and meta-analyses to gain new insights about tinnitus, to develop an evidence-based treatment protocol for effective personalized medicine and to identify new innovative treatment approaches.

## ESIT management and project details

**Figure 1:**
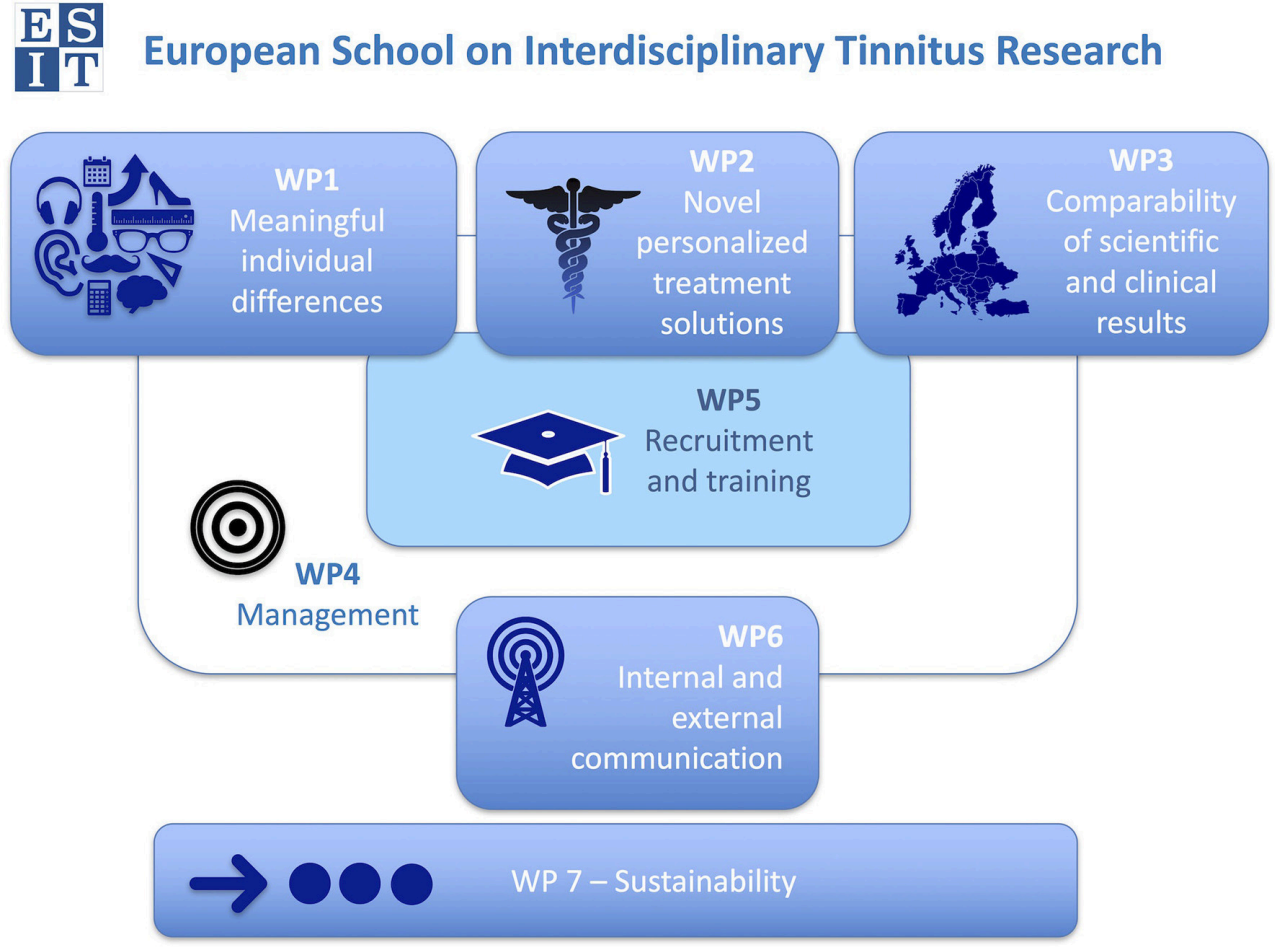
The ESIT structure. Work packages 1–3 deliver the research projects, Work package 4 is to ensure the fluent and effective management of ESIT, Work package 5 is to coordinate the training and recruitment of the students, Work package 6 manages the internal and external communication, and Work package 7 ensures the sustainability of the ESIT achievements. Work packages 4–7 are led by the University of Regensburg, University of Nottingham, Karolinska Institute in Stockholm, and University of Regensburg, respectively.

The ESIT project started on April 1, 2017 with an overall duration of 4 years. The 15 PhD start their work in October 2017. Each student receives funding for 3 years to undertake their PhD. In Figure 1 we outline the work packages of the ESIT project. The first three work packages focus on the scientific goals, while work packages 4–7 concentrate on the effective management of the project (i.e., governance, recruitment and training, communications, and longer-term sustainability). The ESIT office is led by Schlee and based at the department of psychiatry and psychotherapy at the University Clinic Regensburg. More details about the ESIT project and the most recent achievements of the project can be found on the project website (www.esit.tinnitusresearch.net).

## ESIT's innovative perspective on tinnitus research

ESIT is an EU-funded Marie Skłodowska-Curie Innovative Training Network with 12 top-level research institutions in 10 European countries, supporting 15 PhD projects. The research performed under ESIT will be geared toward a more personalized medicine approach, with improved diagnosis and selection of the best-suited therapy based on the individual patient profile. The 15 PhD projects will collectively address three major objectives that are managed under three research-specific work packages. In total, 2 clinics, 8 commercial enterprises, 2 patient organizations, 5 partner academic institutions, and 2 non-profit organizations are actively partnering in the projects and will support 2–3 month research secondments. Secondments are research visits or practical trainings with other academic institutions or industry partners to promote inter-sectoral exchange and embed a broader perspective into the research collaboration from the outset. Commercial partners will also share technological innovations for research purposes.

**Research-specific work package 1. “meaningful individual differences”** Co-ordinated by Lopez-Escamez, work package 1 seeks to determine meaningful individual differences in tinnitus by integrating knowledge and experience from all relevant clinical and scientific disciplines, and combining it with patient-centered and commercial perspectives. This will be achieved through interdisciplinary ESIT partnerships with the Meniere's Disease Society (patient organization), Tinnitus Research Initiative Foundation (non-profit research organization), Sensorion Pharmaceuticals (commercial sector), Julius Maximilian's University Würzburg, and the Knowledge Management and Discovery Laboratory at the Otto von Guericke University. Through this work package:

1a) ESIT will create a conceptual framework, as an end goal, that describes an individual's tinnitus profile. This framework will be based on multi-disciplinary data deposited in a centralized ESIT database that then enables the integration of common data variables gathered from all ESIT projects and data analyses targeted toward informing the tinnitus profile framework.
1b) ESIT will assess the genetic contribution to the development and maintenance of specific subtypes of tinnitus. This work will focus on whole-exome sequencing of people with tinnitus who do not have any detectable otological comorbidities, and of patients with Meniere's Disease and tinnitus.
1c) ESIT will identify factors of the individual tinnitus profile, which affect the general responsiveness to tinnitus treatment.

**Research-specific work package 2. “novel personalized treatment solutions”** Co-ordinated by Weisz, work package 2 seeks to develop novel personalized treatment solutions that respect each patient profile and integrate them with state-of-the-art technological innovations. This will be achieved through interdisciplinary ESIT partnerships with Sivantos, Cochlear Europe, Bee Group AG and Soterix Medical Inc. (commercial sector), and Del Bo Technologia per l'ascolto (independent clinic). Through this work package:

2a) ESIT takes advantage of recent technical developments to develop new, innovative treatment strategies by assessing the effectiveness of enhanced amplification or notched amplification around the frequency corresponding to the dominant tinnitus pitch, individual tomographic neurofeedback, and pseudo-monophasic extra-cochlear stimulation, and by identifying the best placement for an intracochlear microphone.
2b) ESIT seeks to improve existing, clinically well-established treatments by assessing Cognitive Behavior Therapy modified by classical learning, individualized transcranial electric stimulation, repetitive transcranial stimulation combined with auditory stimulation, and an optimized protocol for extra-cochlear electric stimulation.
2c) Using meta-analysis techniques, ESIT will identify the parameters of the individual tinnitus profile which optimize responsiveness to a particular treatment and will use these findings to develop a treatment guide.

**Research-specific work package 3. “comparability of scientific and clinical results”** Co-ordinated by Gallus, work package 3 seeks to promote international comparability of findings across all major relevant disciplines. This will be achieved through interdisciplinary ESIT partnerships with the British Tinnitus Association (patient organization), Brain Products (commercial sector), Nottingham University Hospitals NHS Trust (clinic), and the Institute of Computer Science, University of Tartu. Through this work package:

3a) ESIT will standardize measurements used for diagnostic assessment and inclusion/exclusion criteria for research by developing an evidence-based protocol that will be thoroughly tested using analysis of empirical data, and collecting expert opinions, and patient experiences.
3b) ESIT will integrate large-scale patient data in a central database to support descriptive and inferential analyses.
3c) ESIT will create a standardized framework for collecting longitudinal tinnitus data by implementing novel methods for mobile data collection together with standard assessment tools for validating the crowd-sensing data.
3d) ESIT will collect population-based epidemiological data across many EU countries using the same standardized questions and response options.

This brief overview of the research projects illustrates how the ESIT consortium is characterized by interdisciplinary partnerships both within and across research-specific work packages 1–3. Many projects benefit from the exchange of measurement tools, data sharing, intersectoral knowledge transfer, and dissemination. The overall research program is designed to promote synergies between projects.

## ESIT's innovative perspective on tinnitus training

**Figure 2:**
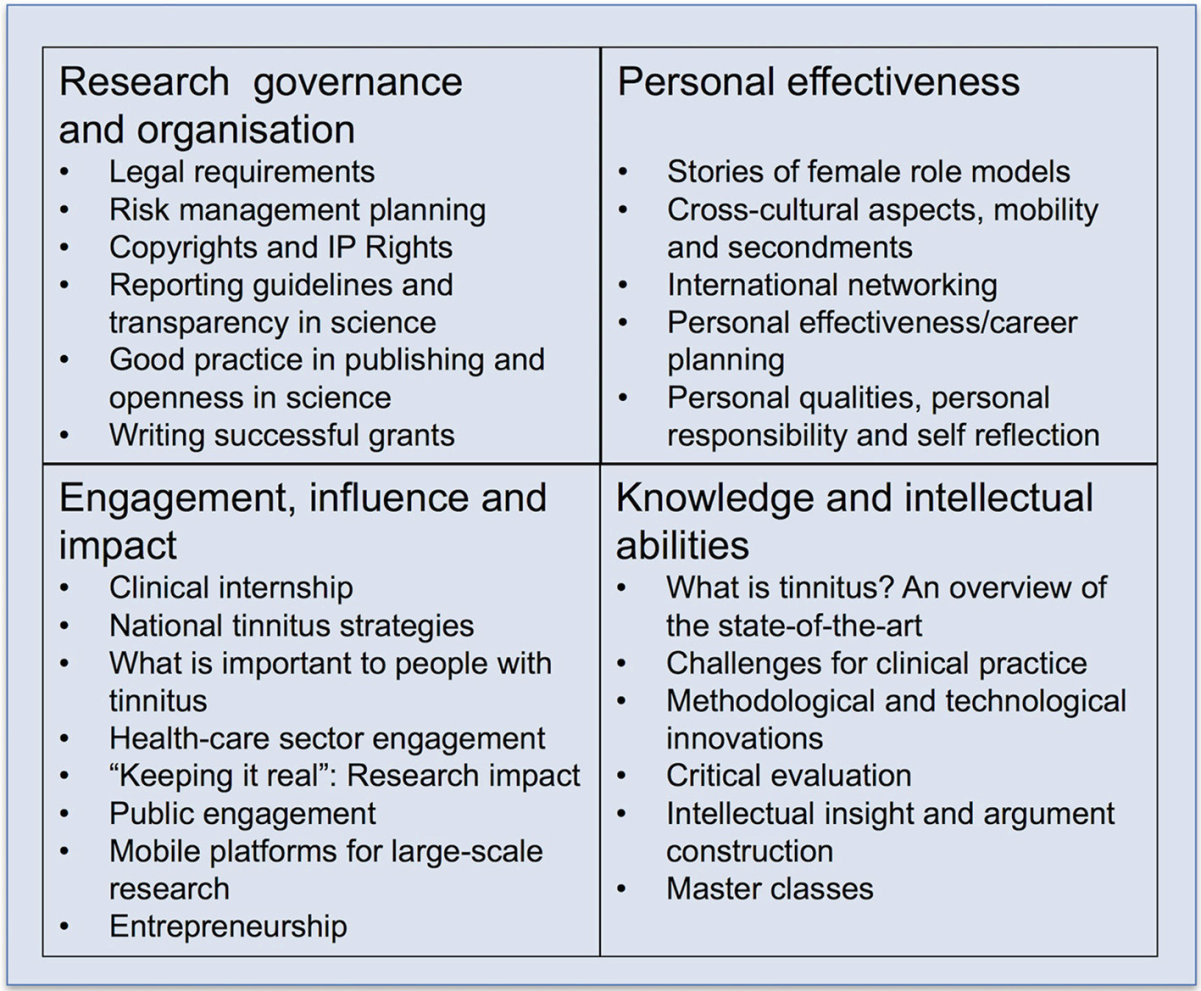
Overview of the ESIT training curriculum structured according to the Vitae Researcher Development Framework. The four essential domains of learning and development are given in the four squares, with individual training modules arranged below.

ESIT provides the first specialized doctoral curriculum on the topic of tinnitus. Acknowledging that “twenty-first century skills” require more than just knowledge building, ESIT PhD students will develop a balance of transferable competencies, as well as tinnitus-specific knowledge and skills (news feature 2015). In this sense it is unique. Each ESIT PhD student will actively manage his/her own dynamic Personal Career Development Plan. To do this we will promote the Vitae Researcher Development Framework Planner, which is a web-based application for mapping professional development (Vitae, 2017). The planner can be used to keep a unified record of all professional development activities, identify their current expertise and capabilities, record learning and development goals and monitor progress, and upload files such as CVs, conference details, and testimonials to record their personal achievements for lifelong learning. The planner uses the Vitae Researcher Development Framework, which considers four essential domains of learning and development and subdomains which inform the learning objectives of the ESIT training modules (Figure 2).

1. **Knowledge and intellectual abilities** To be able to work at the highest level, ESIT PhD students will gain a foundation-level, tinnitus-specific knowledge base, as well as exposure to critical evaluation, intellectual insight, and argument construction. All ESIT PhD students will receive scientific training in the structural and functional components of tinnitus, common diagnostic procedures, and the latest developments in technological innovations for tinnitus care.
2. **Personal effectiveness** Action learning opportunities will foster the personal qualities and approach necessary to become an effective researcher.
3. **Engagement, influence, and impact** Interactions with partners from across the clinical, charity, and commercial sectors will enhance the knowledge and skills that are important for working with others and ensure the wider impact of research.
4. **Research governance and organization** A series of lectures and workshops led by experts in the field will inform students about the standards, requirements, and professionalism to do research.

ESIT offers an innovative doctoral training program (see Figure 2) for ambitious young researchers and provides those PhD students with a set of unique academic, clinical, charity, and commercial sector experiences as well as learning opportunities that extend far beyond any typical academic training program. In the same way that all beneficiaries and most partner organizations are contributing to the research projects in work packages 1–3, work package 5 co-ordinated by Hall describes the training curriculum and the input from many of the ESIT consortium members. In total, 10 commercial enterprises, 6 patient and non-profit organizations, 6 partner academic institutions, 1 health authority and 11 independent clinics will provide practical training for the 15 ESIT students. Most training will be provided by ESIT training schools, satellite events at annual conferences and internet-based courses. Self-study will be guided by supervisors and coordinated and monitored by a Supervisory Board.

A prerequisite for impactful research is an awareness of real-world challenges in the tinnitus clinic. At the start of the program, all ESIT PhD students will complete a clinical internship whereby they will experience for 1 week the activities within a tinnitus clinic in a European country. They will meet with many tinnitus patients and tinnitus care providers, develop an appreciation of the symptoms and impact of tinnitus on the lives of real people, learn to understand the care system in the respective country, and critically evaluate the challenges in that system.

## Planned major dissemination activities

Real-world development opportunities are afforded through several major dissemination activities. First, ESIT PhD students will write a special commissioned feature for publication in “ENT & Audiology News” magazine aimed at hearing healthcare professionals. Second, the ESIT network aims for a completely revised and updated 2nd edition of the “Textbook of Tinnitus” (Møller et al., 2011). The revised edition will be tailored toward becoming the number one choice as a textbook for doctoral and medical training in tinnitus. Other dissemination activities include an updated version of the Wikipedia page for “Tinnitus” (Wikipedia, 2017Q12), an educational video on tinnitus prepared in the major European languages, and dissemination of activities through social media channels. Dissemination will be coordinated by Cederroth, leader of the work package 6.

## Impact on career prospects

To enable clinical relevance of academic research and its rapid translation into improved tinnitus healthcare, there is an urgent need for closer intersectoral collaboration. In conclusion, ESIT will achieve this by providing high-level training with a multidisciplinary supervisory team. The program will equip ESIT PhD students not only with a comprehensive understanding of the challenges faced by healthcare providers in treating tinnitus and by industry in providing technological healthcare solutions, but also to lay the foundations for leadership positions in the relevant academic and non-academic sectors. The network-integrated training program will ensure that the ESIT PhD student can effectively communicate between disciplines and will have the skills to identify synergistic opportunities, to build bridges between disciplines to exploit these connections, and to interact successfully with the private sector. Exposure across disciplines and sectors is unique and is essential to advance ESIT student personal career development and lead the students into European leadership positions in the future. In this way, ESIT will create a new generation of tinnitus experts who are sensitive to the issues of heterogeneity, have a broad knowledge base and have first-hand experience of the possibilities for inter-sectoral disciplines.

## Author contributions

WS, DH, and CC drafted the initial version of the manuscript and created the figures. All authors contributed to the development of the ESIT project and contributed equally to all other stages of the manuscript development. All authors approved the manuscript.

### Conflict of interest statement

The authors declare that the research was conducted in the absence of any commercial or financial relationships that could be construed as a potential conflict of interest.
